# Comparison of four DNA extraction kits efficiency for 16SrDNA microbiota profiling of diverse human samples

**DOI:** 10.2144/fsoa-2022-0072

**Published:** 2023-03-09

**Authors:** Sandrine Le Gall-David, Gaëlle Boudry, Sylvie Buffet-Bataillon

**Affiliations:** 1 Institut Numecan, INSERM, INRAE, Univ Rennes, Rennes, France; 2 Bacteriology, Pontchaillou University Hospital, Rennes, France

**Keywords:** 16S rRNA gene sequencing, bacterial DNA extraction, bead-beating, data analysis, DNA binding, enzymatic lysis, human microbiota, Illumina, low biomass, next generation sequencing

## Abstract

**Aim:**

The current study investigated the performance of 4 widely used DNA extraction kits using different types of high (stool) and low biomass samples (chyme, broncho alveolar lavage and sputum).

**Methods:**

Qiagen Powerfecal Pro DNA kit, Macherey Nucleospin Soil kit, Macherey Nucleospin Tissue Kit and MagnaPure LC DNA isolation kit III were evaluated in terms of DNA quantity, quality, diversity and composition profiles.

**Results:**

The quantity and quality of DNA varied among the four kits. The microbiota of the stool samples showed similar diversity and compositional profiles for the 4 kits.

**Conclusion:**

Despite differences in DNA quality and quantity, the 4 kits yielded similar results for stool samples, while all kits were not sensitive enough for low biomass samples.

## Plain language summary

DNA extraction is a major factor affecting the microbial profile of various samples. Considering that different kits are commonly used such as QIAamp PowerFecal Pro DNA kit (QPFPD, QIAGEN), Macherey Nucleospin Soil (MNS, MACHEREY-NAGEL) Macherey Nucleospin Tissue (MNT, MACHEREY-NAGEL) and MagnaPure LC DNA isolation kit III (MPLCD, ROCHE), this study aimed to assess their performance using high (feces) and low-biomass samples. The kits were equally effective for feces samples but not sensitive enough for low biomass samples (chyme, bronchoalveolar lavage fluid and sputum).

## Background

A paradigm shift in the origin of many human diseases occurred 15 years ago when the role of the different microbiotas inhabiting the host (intestinal, vaginal, skin, etc.) on its own physiology and in the pathophysiology of numerous diseases has emerge [1]. The term ‘human microbiota’ has gained enormous attention in research since then, reaching more than 32,000 articles in Pubmed in the last 5 years. Indeed, the drop in sequencing cost and the development of bioinformatical tools simplified the analysis of microbiota for many laboratories. Several protocols for sampling, DNA extraction, sequencing, and bioinformatic analysis are now available. However, this multitude of protocols does not allow easy comparisons between studies nor the pooling of data in an open-science strategy.

One significant cause of variability between studies analyzing microbiota through amplicon sequencing is DNA extraction process, next after sample type and origin and storage methods [2,3]. Several kits are available on the market, whose protocols share similar pipelines: lysis of bacteria through mechanical, chemical or enzymatic methods, binding of DNA on columns, several wash steps to remove proteins and other contaminants and final elution. The type of bacterial wall disruption technique can affect DNA yield, quality of DNA as mechanical lysis can disrupt all types of bacteria but likely fragment genomic DNA [4]. On the other hand, enzymatic and chemical lysis methods are less efficient to lyse bacteria and might result in under-estimation of tough-to-disrupt bacteria, yet preserving DNA quality [5]. DNA binding column characteristics and washing solution composition also differ between kits, resulting in different DNA yield and purity.

In recent years, more attention has been given to the standardization of the workflow of microbiome research [2,6,7]. However, different internal extraction kits and methods are used in different laboratories. Recently, Costea *et al.* evaluated 21 methods for extracting DNA from three continents and suggested a protocol, called the Q protocol, as the gold standard for human stool samples [6]. They reported that it was not known if this method was optimal for samples other than fecal matter, such as low biomass samples.

Our objective was therefore to assess the efficiency of 4 commercially DNA extraction kits on bacterial DNA quantity, quality and to analyze the impact on bacterial composition and diversity of different samples. We checked DNA extracted with those methods quantity and quality and performed 16SrDNA sequencing using Illumina technology followed by taxonomic assignment to analyze differential abundance and alpha and beta diversity.

## Methods

### Sample collection

**Figure 1.**
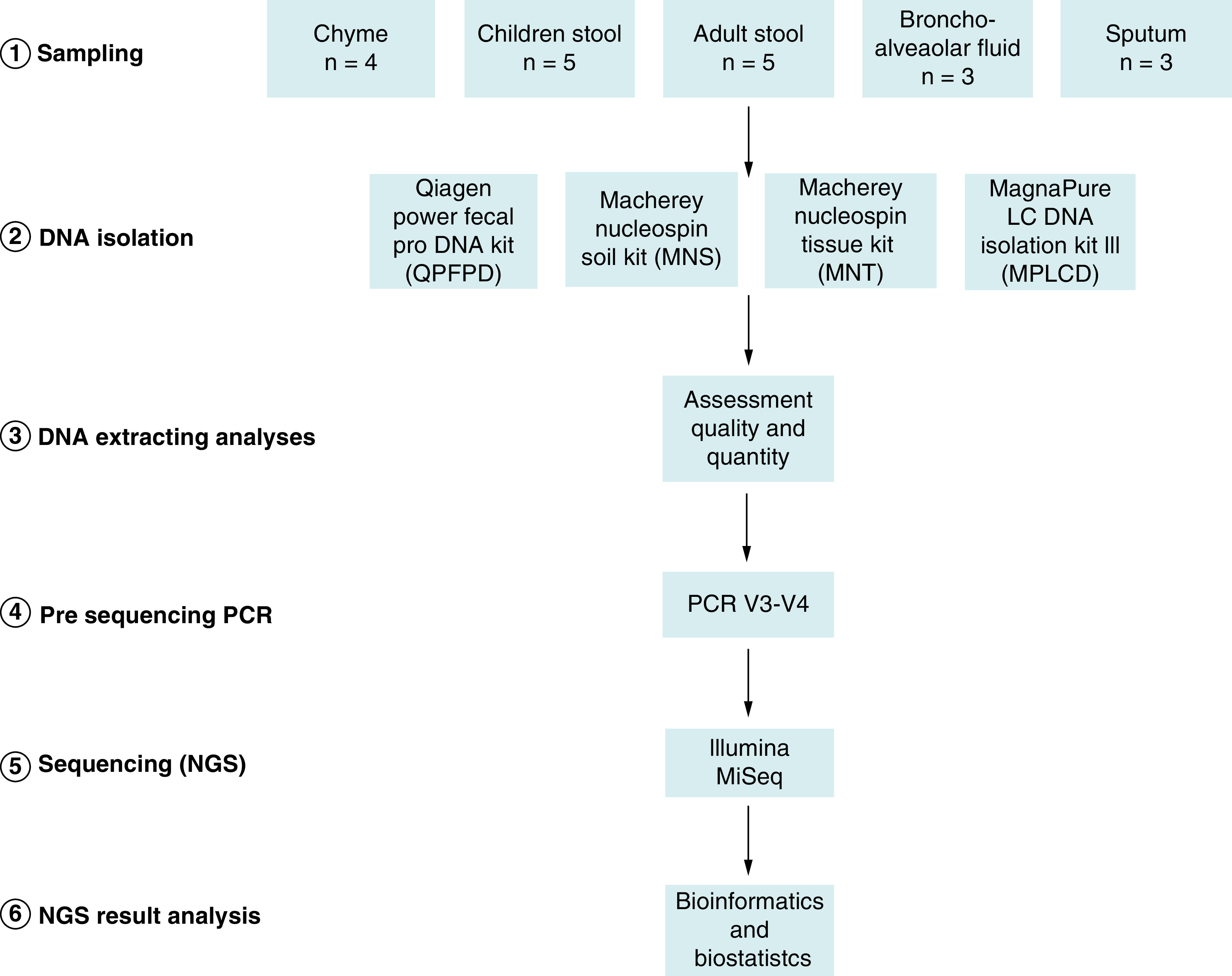
Experimental design. Schematic representation of procedures and conditions for extraction and analysis of bacterial DNA from 5 different human sample types. Five different types of samples from different collection sites or from different ages **(1)** were extracted with 4 different DNA extraction methods **(2).** DNA quality and quantity was assessed **(3)**, then the V3-V4 16S rDNA amplified **(4)** and sequenced **(5)** and analyzed **(6).**

The outline of the study design is presented (Figure 1). Samples were divided in five groups from different collection sites which differed in term of microbiota density: chyme (mix of gastric juice and partly digested food passing from the stomach to the small intestine [n = 4]), feces (n = 5 from infants; n = 5 from adults), bronchoalveolar lavage fluid (BAL) (n = 3), sputum (n = 3). Samples were collected following the recommendations of the International Human Microbiome Standards (IHMS) (www.microbiome-standards.org). When received at the laboratory, BAL and sputum samples were centrifuged for up to 10 minutes at 8,000 × g to concentrate the bacterial cells, discard most of the supernatant, leaving only leaving only 200 μl. Fecal samples were manually homogenized and weighed into separate aliquots. Then all the samples were stored at 80°C until DNA extraction.

### DNA extraction

Samples were processed using four commercial DNA isolation kits. QIAamp PowerFecal Pro DNA kit (QPFPD, QIAGEN), Macherey Nucleospin Soil (MNS, MACHEREY-NAGEL) Macherey Nucleospin Tissue (MNT, MACHEREY-NAGEL) and MagnaPure LC DNA isolation kit III (MPLCD, ROCHE) were used following manufacturers' instructions for each kit. A peanut-sized stool samples (∼200 mg) suspended in 100 μl of PBS, 200 μl of liquid samples (BAL and sputum) and 500 μl of chyme were used for each extraction.

#### QPFPD

Each sample as described before was introduced in bead-containing PowerBeads Pro Tubes with lysis buffer. These mixtures were subjected to mechanical lysis for 5 minutes. Different buffers were added before transferring the mixture to MB Spin Columns and centrifuged. After different washing steps of the column, DNA was eluted with 100 μl C6 buffer and stored at -80°C.

#### MNS

In this method, each sample were introduced in tubes containing type A beads. To lyse each sample and precipitate contaminants, different buffers were added to samples. These mixtures were subjected to mechanical lysis for 5 minutes. Obtained lysate was then filtered and DNA bind to a NucleoSpin Soil column. After a few washes of the column, DNA was eluted with 100 μl SE buffer and stored at -80°C.

#### MNT

This extraction kit uses proteinase K for lysis. Proteinase K solution was added to each sample which was then thoroughly mixed and incubated at 56°C during 1 h then at 70°C during 10 min. To precipitate DNA, ethanol 100% was added and mixture were subjected to mechanical lysis for 5 minutes. To bind DNA, lysate was load onto a NucleoSpin Tissue column. After a few washes of the column, DNA was eluted with 100 μl BE buffer and stored at -80°C.

#### MPLCD

In this DNA extraction kit proteinase K was used, associated with lysis buffer. This buffer was added to each sample and samples were incubated for 10 min at 65°C and another 10 min at 95°C. To homogenize these lysates, they were subjected to mechanical lysis for 30 s. Lysate were then transfer in the wells of the Sample Cartridge, each well contains magnetic-bead. DNA bind to these Magnetic Glass Particles (MGPs). Wash Buffer is then used to wash MGPs with bound DNA to remove unbound substances. Purified DNA was eluted with a 100 μl of low salt elution buffer and stored at -80°C.

Each kit had different types of cell lysis and DNA binding methods as summarized in Table 1.

**Table 1.** Characteristics of DNA isolation kits used in this study.

Full name of the kit	Kit name abbreviation	Mechanical cell lysis (bead-beating)	Enzymatic lysis	Chemical lysis	Heat treatment	DNA binding
QIAamp PowerFecal Pro DNA kit	QPFPD	Yes	No	Yes	No	Silica membrane-based columns
Macherey Nucleospin Soil	MNS	Yes	No	Yes	No	Silica membrane-based columns
Macherey Nucleospin Tissue	MNT	No	Yes (PK)	Yes	Yes	Silica membrane-based columns
MagnaPure LC DNA isolation kit III	MPLCD	No	Yes (PK)	Yes	Yes	Magnetic beads

MNS: Macherey Nucleospin Soil; MNT: Macherey Nucleospin Tissue; MPLCD: MagnaPure LC DNA isolation kit III; PK: Proteinase K; QPFPD: QIAamp PowerFecal Pro DNA kit.

### Assessment of quantity & quality of DNA

#### DNA concentration

The concentration of DNA obtained after the different extraction procedures for each sample was quantified using a Qubit 3.0 fluorometer with the Quant-iT dsDNA HS Assay (assay range between 0.2 and100 ng; sample concentration between 10 pg/μl and 100 ng/μl) and the Quant-iT dsDNA BR Assay (assay range between 2 and 1000 ng; sample concentration between 100 pg/μl and 1000 μg/μl), according to manufacturer's instructions (ThermoFischer Scientific).

#### DNA quality

DNA quality was determined using a NanoDrop ND-1000 [8] after calibration according to the NanoDrop 2000–2000c & 1000 Calibration Check procedure (ThermoFisher Scientific). The optical density (OD) ratio between 260 nm and 280 nm was used to asses acidic pH or contamination (proteins, phenol, other impurities) for samples with a ratio below 1.5–2.0. The OD 260 nm/230 nm was also used to check contamination by polysaccharides, phenol or low pH [8,9] for samples with a ratio below 1.75.

DNA quality was also determined on 1 μl of total DNA using Agilent 4200 Tape Station (Agilent), which separates DNA fragments by size on a chip. Laser induced fluorescence is then used to translate bands in peaks to evaluate the distribution and relative amounts of DNA of different sizes.

### DNA sequencing

The V3–V4 region of the 16S rRNA gene was amplified by PCR using F343 and R784 primers (F343: CTTTCCC TACACGACGCTCTTCCGATCTTACGGRAGGCAGCAG, R784: GGAGTTCAGACGTGTGCTCTTCCGATCTTACCAGGGTATCTAATCCT) as previously described [10]. Briefly, purified genomic DNA (gDNA) was amplified with the primers F343 and R784 using 30 amplification cycles with an annealing temperature of 65°, producing amplicons of 510 bp, although the exact length varies depending on the organisms. Extremely high-quality, full-length reads of the entire V3 and V4 region were generated thanks to the overlapping of the MiSeq paired 250-bp reads, in a single run. Home made 6 bp index added to R784 during a second PCR (12 cycles, forward primer (AATGATACGGCGACCACCGAGATCTACACTCTTTCCCTACACGAC) and reverse primer (CAAGCAGAAGACGGC ATACGAGAT-index-GTGACTGGAGTTCAGACGTGT) was used to perform single multiplexing After purification of the PCR products, they wereloaded onto the Illumina MiSeq cartridge according to the manufacturer instructions. PhiX was used to check the quality of the run internally., Each pair-end sequences were assigned to its sample thanks to the previously integrated index. Flash software [11] was used to assemble each pair-end sequences using at least a 10 bp-overlap between the forward and reverse sequences, allowing 10% of mismatch. These sequencing and cleaning steps were performed at the GeT-PlaGE platform (Toulouse, France) as described by Zemb *et al.*, 2020 [12].

The resulting sequences were processed using QIIME2 pipeline (v. 2018.4, https://qiime2.org/) after importation of reads as “PairedEndFastqManifestPhred33” format, read denoising with DADA2 and truncation of the forward and reverse sequences at 240 bases and all other parameters set to default. Chimeric sequence were detected, non-16S rDNA gene identified n, and amplicon sequence variants (ASVs) with a similarity threshold of 99% clustered using the SILVA (release 132) reference database [13,14]. ASVs with a total frequency <10 in the entire table and present in only 1 sample were removed, as well as reads classified as Archaea or Unassigned.

After cleaning and filtering of the reads, only the samples corresponding to the stools could be analyzed since too few reads passed the quality controls for other samples.

The core diversity analysis was performed with QIIME2 diversity core-metrics-phylogenetic plugin, with a specific sampling depth, 5900 reads for the children stool samples and 6800 reads for the adult stool samples.

### Statistical analysis

To assess the influence of DNA extraction kits on DNA quantity, quality (i.e., A260/280 and A260/230), Chi2 or Kruskall-Wallis tests were perfomed. Species richness (Chao1) and evenness (Shannon index) were calculated for alpha diversity estimations and compared using Kruskal-Wallis tests. Permutational multivariate analysis of variance (PERMANOVA with the Adonis function) on beta diversity distance matrices (Bray Curtis, Unweighted Unifrac) was used to evaluate the impact of the 4 different extraction kits on microbiota composition variability between samples. A P-values <0.05 was considered significant. To compare the taxonomy composition between different extraction kits Kruskall-Wallis or Wilcoxon tests were performed.

## Results

### Influence of DNA extraction kits on the quantity & quality of DNA

**Figure 2.**
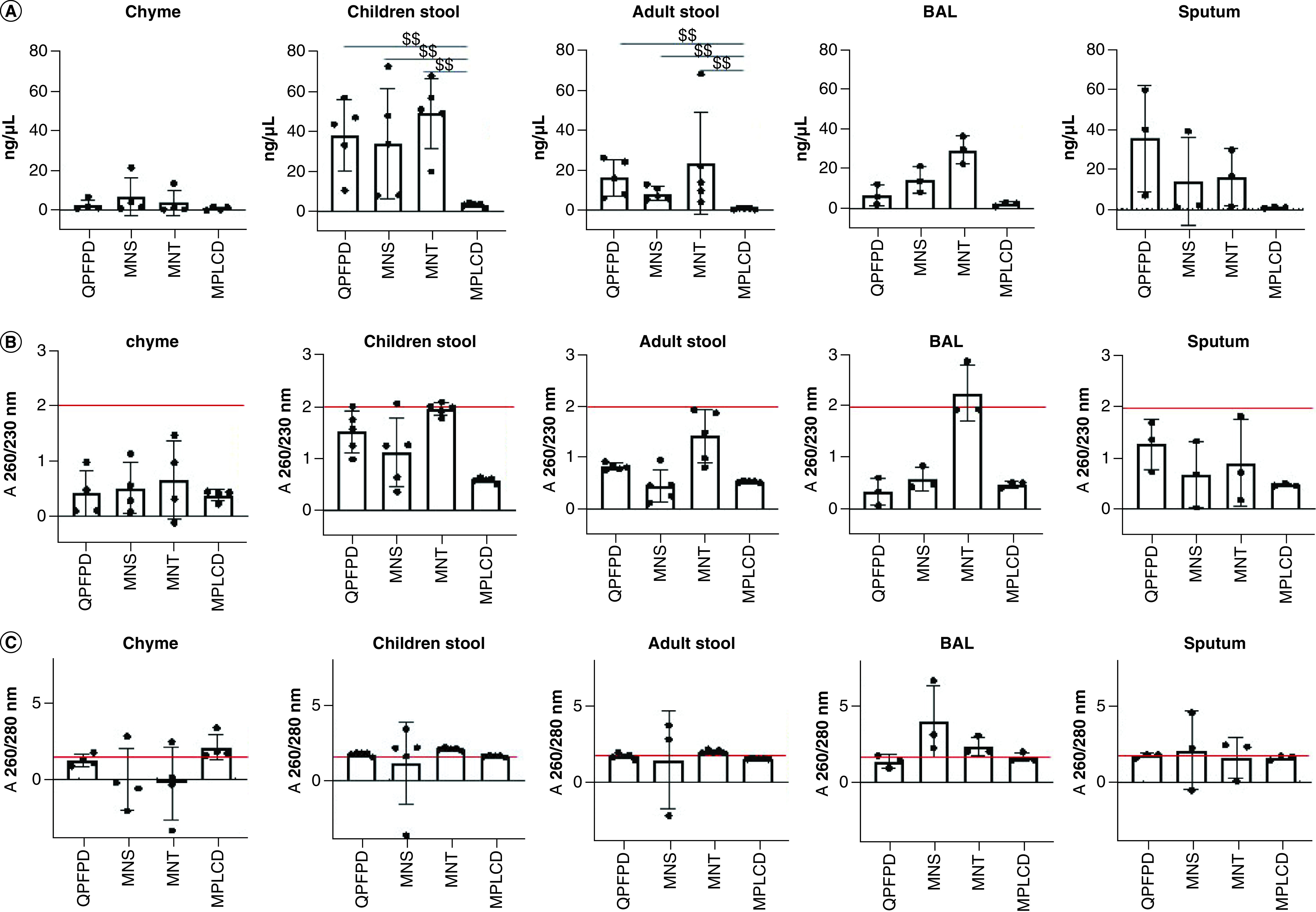
Concentration and quality of extracted DNA according to DNA extraction kits. **(A)** DNA concentration using the Qubit device. **(B)** Nanodrop 230/260 ratio. **(C)** Nanodrop 260/280 ratio. The red line corresponds to the optimum ratio for DNA quality. MPLCD resulted in the lowest DNA yield compared with the others kits. Chyme samples had very low DNA concentration. MNT was the kit that produced the highest percentage of DNA extracts with a A260/A230 ratio greater than 1.75. All the samples showed an A260/A280 ratio greater than 1.5, except for chyme samples extracted with the MNS, MNT and QPFPD kits and BAL samples extracted with the QPFPD kit. ^$$^p < 0.01. MNS: Macherey Nucleospin Soil; MNT: Macherey Nucleospin Tissue; MPLCD: MagnaPure LC DNA isolation kit III; QPFPD: QIAamp PowerFecal Pro DNA kit.

**Figure 3.**
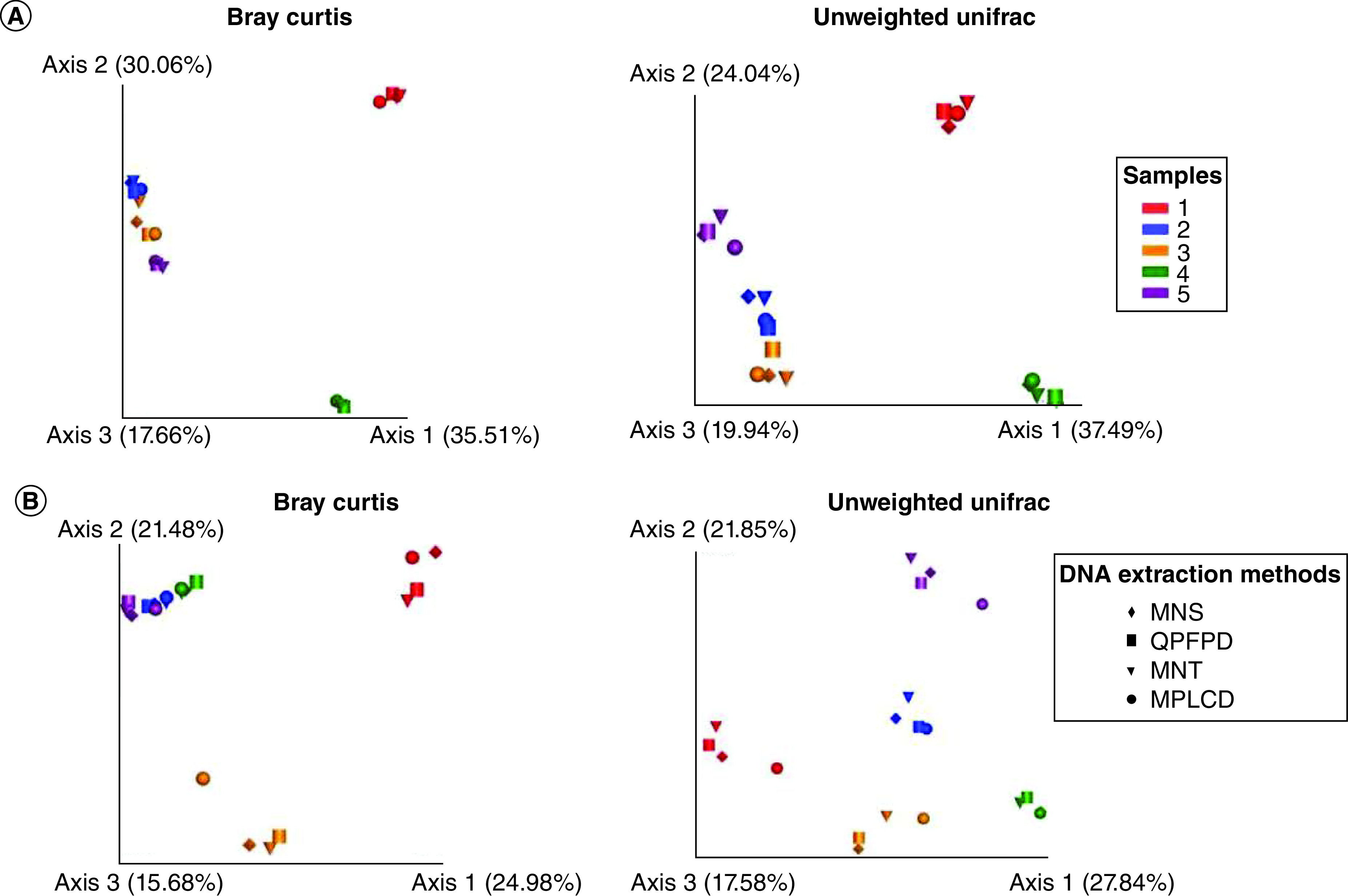
Impact of DNA extraction kits on beta-diversity of fecal microbiota. Principal coordinates analysis (PCoA) based on beta diversity distances (Bray–Curtis dissimilarity and unweighted Unifrac similarity) of different stool samples extracted with different DNA extraction kits.**(A)** Children stools. **(B)** Adult stool samples). PcoA of beta-diversity did not reveal observable clusters according to according to the method, using both the Bray Curtis and unweighted UniFrac distance. MNS: Macherey Nucleospin Soil; MNT: Macherey Nucleospin Tissue; MPLCD: MagnaPure LC DNA isolation kit III; QPFPD: QIAamp PowerFecal Pro DNA kit.

Pairwise comparisons indicated that the MPLCD kit yielded the lowest amount of DNA (p < 0.01, Figure 2) for stool samples (children and adult) compared with the other kits. No significant difference between extraction kits was observed for the other samples. Noteworthy, DNA concentration was very low for non-stool samples, especially for chyme ones (<10 ng/μl), irrespective of the extraction kit (Figure 3).

The impact of extraction kits on DNA quality was then assessed via the measurement of A260/A280 and A260/A230 ratios using a Nanodrop or via fragment size distribution using a Bioanalyzer. Nearly all the samples exhibited an A260/A280 ratio greater than 1.5, which is indicative of relatively pure DNA, except for chyme samples extracted with the MNS, MNT and QPFPD kits and BAL samples extracted with the QPFPD kit (Table 2).

**Table 2.** Percentage of samples with A260/280 ≥1.5.

	Chyme (n = 4)	Children stool (n = 5)	Adult stool (n = 5)	Broncho alveolar lavage fluid (n = 3)	Sputum (n = 3)
QPFPD	25	100	80	33	100
MNS	0	80	60	100^†^	67
MNT	25	100	100^‡^	100^†^	67
MPLCD	100^†^^,^^‡^^,^^§^	100	80	67	100

Within a column:

† p < 0.05 vs QPFPD.

‡ p < 0.05 vs MNS.

§ p < 0.05 vs MNT. Nearly all the samples exhibited an A260/A280 ratio greater than 1.5, except for chyme samples extracted with the MNS, MNT and QPFPD kits and BAL samples extracted with the QPFPD kit.

MNS: Macherey Nucleospin Soil; MNT: Macherey Nucleospin Tissue; MPLCD: MagnaPure LC DNA isolation kit III; QPFPD: QIAamp PowerFecal Pro DNA kit.

MNT was the kit that produced the highest percentage of DNA extracts with a A260/A230 ratio greater than 1.75, indicative of less residual carryover compared with the other kits (Table 3). Noteworthy, all the DNA extracts produced with the MPLCD kit exhibited a A260/A230 ratio below 1.75, irrespective of the type of samples. Whatever the DNA extraction kit used, all the A260/230 ratio of DNA extracts from chyme samples were under 1.75.

**Table 3.** Percentage of samples with A260/230 ≥1.75.

	chyme (n = 4)	children stool (n = 5)	adult stool (n = 5)	Broncho alveolar lavage fluid (n = 3)	sputum (n = 3)
QPFPD	0	40^§^^,^^¶^	0	0	0
MNS	0	20^§^	0	0	0
MNT	0	100^†^^,^^‡^^,^^¶^	40^†^^,^^‡^^,^^¶^	100^†^^,^^‡^^,^^¶^	33
MPLCD	0	0^†^^,^^§^	0	0	0

Within a column:

† p < 0.05 vs QPFPD.

‡ p < 0.05 vs MNS.

§ p < 0.05 vs MNT.

¶ p < 0.05 vs MPLCD. With the exception of chyme samples, the DNA extracts produced with the NMT kit mainly had a A260A230 ratio higher than 1.75. All DNA extracts, with the exception of chyme samples produced with the MPLCD kit, had an A260/A230 ratio lower than 1.75, regardless of the type of sample.

MNS: Macherey Nucleospin Soil; MNT: Macherey Nucleospin Tissue; MPLCD: MagnaPure LC DNA isolation kit III; QPFPD: QIAamp PowerFecal Pro DNA kit.

Stool samples were analyzed with a Bioanalyzer DNA high-sensitive chip (Agilent), which provides information on DNA fragment size distribution. Bioanalyzer images of the libraries showed a homogenous smear of DNA from 1500 to 10000 bp for the QPFPD, MNS and MNT extraction kits (data not shown). For MPLCD, all samples showed a similarly average size <500 pb (data not shown). Other samples (chyme, BAL and sputum) were not analyzed due to the poor quantity of the DNA extracted.

### Influence of DNA Extraction Kits on Microbial Diversity & Composition

#### Diversity Analysis

A total number of 609,773 raw sequences was obtained, varying from 0 to 23,136 sequences by samples (Table 4). The number of reads for chyme, sputum and BAL samples was generally very low. Therefore, no core metrics analyze was possible and no data are presented for these samples. To determine if DNA extraction kits impact beta diversity, PCoA plots were constructed based on Bray–Curtis and unweighted UniFrac distance matrices to assess if samples clustered together or not. As shown in Figure 3A & B, samples clustered according to the individual and not the DNA extraction kits. Beta-diversity analyses did not show any significant sample clustering based on extracted method (p > 0.05) (Table 5).

**Table 4.** Number of reads obtained after V3-V4 16rDNA sequencing.

	chyme (n = 4)	children stool (n = 5)	adult stool (n = 5)	Broncho alveolar lavage fluid (n = 3)	sputum (n = 3)
QPFPD	0 [0–0]	12,517 [9,661–17,932]	12,981 [10,073–16,137]	5,384 [0–13,102]	79 [14–4,757]
MNS	0 [0–226]	13,560 [8,401–23,136]	13,589 [8,587–18,474]	6,976 [3,126–9,910]	87 [53–14,465]
MNT	0 [0–148]	12,548 [8,716–13,098]	18,983 [6,886–22,182]	2,266 [825–10,776]	155 [106–3,423]
MPLCD	397 [11–2154]	10,321 [5,694–15,650]	14,125 [9,678–17,042]	1,660 [0–7,146]	186 [0–3,423]

Median and [min–max] number of reads obtained by sample. An overall number of 609,773 raw sequences were obtained, ranging from 0 to 23,136 sequences per sample. The number of reads for chyme, spitting and BAL samples was very low.

MNS: Macherey Nucleospin Soil; MNT: Macherey Nucleospin Tissue; MPLCD: MagnaPure LC DNA isolation kit III; QPFPD: QIAamp PowerFecal Pro DNA kit.

**Table 5.** Results (P-value) of the permutational multivariate analysis of variance.

	Extraction kit effect		Sample effect	
	Bray Curtis	Unweighted Unifrac	Bray Curtis	Unweighted Unifrac
**Children stool**	1	0.99	0.003	0.001
**Adult stool**	0.98	0.72	0.001	0.001

PCoA analyses based on the Bray–Curtis index, unweighted Unifrac distance method with PERMANOVA as statistical methods were conducted to reveal the variation of the microbiota between the four kits. Bray–Curtis and unweighted UniFrac analyses exhibited no significant differences in microbiota composition between the 4 kits (p > 0.05).

**Figure 4.**
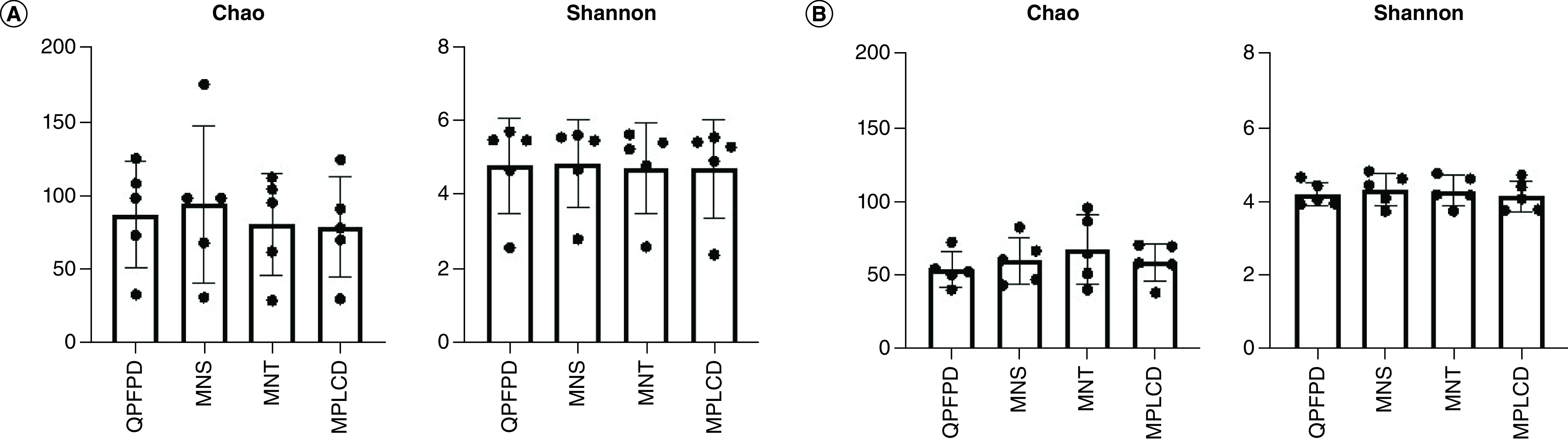
Impact of DNA extraction kits on alpha diversity. Chao1 and Shannon indexes were calculated and compared for children **(A)** and adult **(B)** stools. No difference was found among the different DNA extraction kits for both alpha-diversity metrics. MNS: Macherey Nucleospin Soil; MNT: Macherey Nucleospin Tissue; MPLCD: MagnaPure LC DNA isolation kit III; QPFPD: QIAamp PowerFecal Pro DNA kit.

Furthermore, we assessed the impact of the different extraction kits on alpha-diversity using the Chao and Shannon indices. No difference was found among the different DNA extraction kits for both alpha-diversity metrics (p > 0.05, Figure 4).

#### Taxonomic Identification

**Figure 5.**
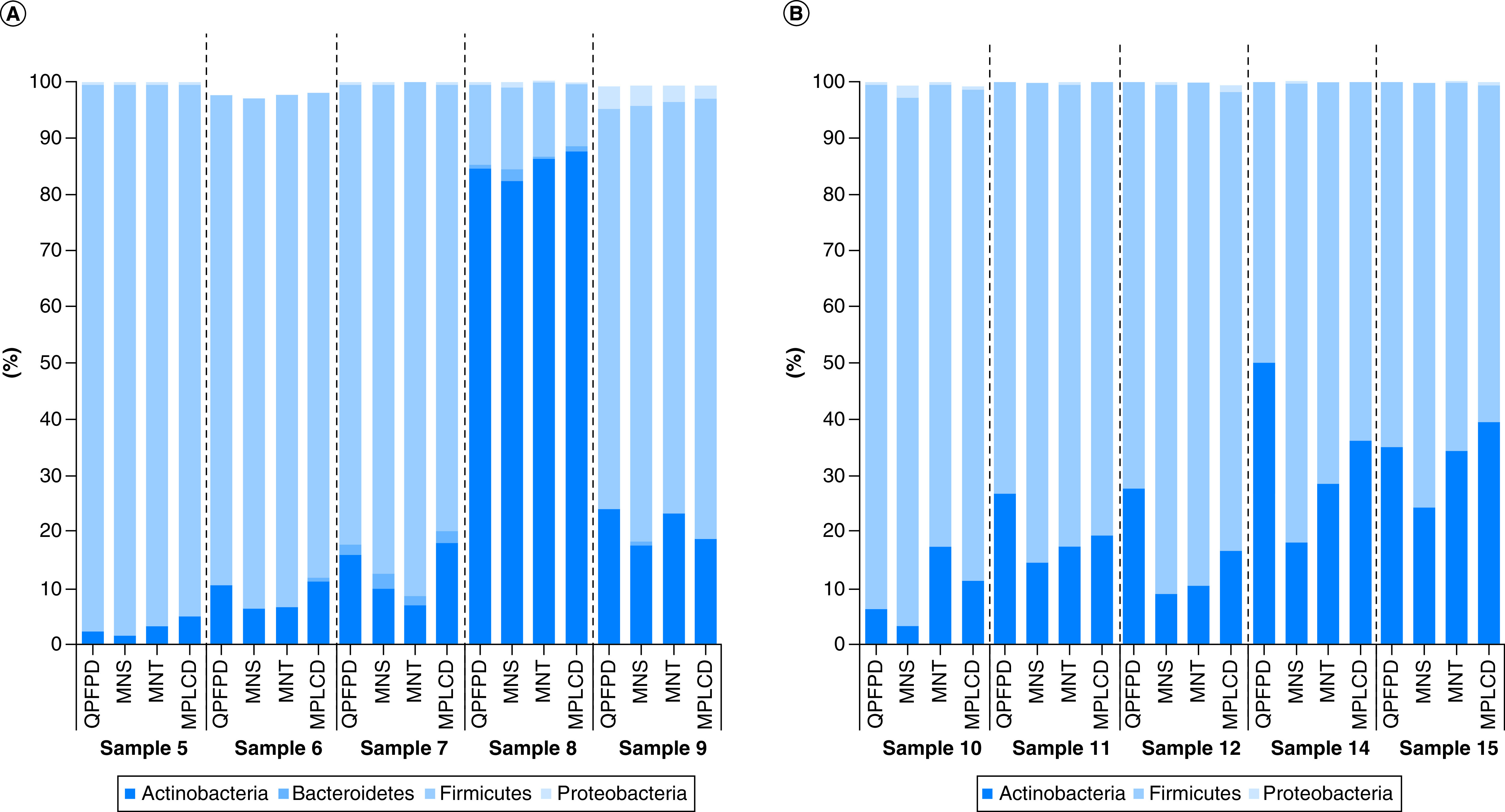
Comparison of four DNA extraction kits on phylum relative abundance after 16S rDNA sequencing. Phylum relative abundance of children **(A)** and adult **(B)** stools extracted with different DNA extraction kits. No significant differences were observed across any of the 4 methods.

Taxonomic compositions of the microbiota were compared with evaluate if the different DNA extraction kits affected community composition. When annotated at the phylum, family or genus levels, the microbial profile detected in children and adult stools demonstrated no significant differences between QPFPD, MNS, MNT and MPLCD (p > 0.05, Figure 5).

## Discussion

Our objective was to asses if the type of DNA extraction kits used to extract DNA before 16S rDNA sequencing can impact microbiota diversity and composition analysis of diverse human samples. We used 4 commercially available kits to extract DNA from low microbiota concentration samples (sputum, BAL, chyme) or high microbiota concentrations ones (children and adult stools) to compare their efficiency in terms of DNA yield, DNA quality and microbiota diversity after sequencing. The 4 commercially available kits used in this study extracted bacterial DNA with some variabilities. Among the 4 kits, MPLCD was the less efficient in terms of amount and quality of DNA, whereas the other 3 kits resulted in a better quantity and quality of DNA.

The DNA extraction kits tested differed in terms of lysis technique (mechanical lysis and/or chemical lysis and/or heating) and DNA binding technique (columns vs magnetic beads). QPFPD and MNS both involves bead beating while MNT and MPLCD relies on proteinase K and mechanical (vortex) lysis and heating. Among factors contributing to DNA quantity and quality variations when extracting microbial nucleic acids, lysis technique seems a key step [15,16]. However, the literature is still controversial. Indeed, while only few significant differences between DNA extraction methods when assessing the role of lysis technique were observed by Claassen *et al.*, Yuan *et al.* reported that bead beating and/or mutanolysin was the most effective cell lysis and DNA recovery technique [17,18]. A combination of lysing procedures seems a good option to capture the most accurate community composition since hot phenol and bead beating were described as the most effective lysing procedure in another study [19]. A bead-beating step during DNA extraction has been shown to be efficient to break down the cell wall of Gram-positive bacteria [20]. QPFPD and MNS uses bead beating as mechanical lysis. By contrast, MPLCD and MNT combine proteinase K buffer and no bead-beating. In our study, the additional bead beating step in QPFPD and MNS did not influence the relative abundance of phyla in these samples, as expected by Gryp *et al.* [21]. Indeed, Gryp *et al.* showed that the Qiagen PowerMicrobiome kit combining bead beating with proteinase K and mutanolysin was more effective than without pre-treatment. The QIAmp DNA Stool Minikit, with a beat-beating step and the QIAmp PowerFecal Pro DNA Kit provided comparable results in terms of DNA quantity and microbiome profiles [22].

The second factor that could have affected DNA yield and quantity is the type of technique used to trap DNA: columns are used in the QPFPD, MNS and MNT kits while magnetic beads are used in the MPLCD kit. Spin Column technology relies on the affinity between negatively charged DNA and positively charged silica material. This results in selective binding of DNA to the silica matrices and wash-out of the rest of the cell components and other chemicals. These methods include steps of lysis then centrifugations for the purpose of DNA binding. DNA is finally eluted from the silica matrix by any hyposmotic solution, such as nuclease-free water in the QPFPD, MNS and MNT kits. The different washing and elution steps are achieved to purify and elute a high amount of nucleic acids as well as to recover small fragments DNA efficiently. This technology has been shown to result in high-purity DNA with great quantitative and qualitative reproducibility [23].

Magnetic beads technology is an alternative way for DNA extraction. This technology is widely used in automated extraction procedures for a large number of samples [24,25]. The technique is based on magnetic particles, whose surface is positively charged and binds DNA at pH ≤6.5. A change in pH to pH ≥8.5 allows the release of the DNA bound to the particles [26]. Functionalized magnetic particle or beads coupled to adequate buffers have been developed to result inefficient DNA extraction. One positive aspect of this technology is the absence of centrifugation steps possibily producing shear forces, causing breaking of nucleic acids.

Even if, MPLCD was the only kit using magnetic beads and yielded the lowest DNA quantity, magnetic bead DNA extraction method is not less efficient in DNA extraction. In accordance with Knudsen and al [27]. DNA concentration is independent of DNA separation types. In their study, the highest DNA concentrations for human feces were obtained using the Easy-DNA (phenol-chloroform precipitation), MagNA Pure (magnetic beads), QIAamp DNA stool minikit (silica membrane- based columns) procedures. On average across the types of specimen (Human, pig Sewage), the highest DNA concentrations were obtained using Easy-DNA (Phenol-chloroform precipitation) and QIAStool (Silica membrane- based columns), and the lowest were obtained using the PowerSoil.HMP (Silica membrane-based columns) and InnuPure (Magnetic beads) methods.

In our study, the extraction kit used did not result in any difference in in microbial community diversity, considering the Shannon or Chao diversity indices. Thus, probably because of the amplification step before 16S rDNA sequencing, the quantity of DNA yielded by the extraction method does not constitute an attention point when choosing a DNA extraction kit. In accordance with Knudsen and al, there was no significant correlation between the amount of DNA obtained and the community diversity or richness measured [27]. Using synthetic consortia, Ducarmon *et al.* showed that the different DNA extraction methods they used allowed them to retrieve the theoretical relative bacterial abundance but with, differences according to the methods they used, while little variation were seen with the type of library preparation and sequencing they tested. Moreover, the bioinformatic pipelines they used resulted in different results for observed richness, but diversity and compositional profiles were comparable [28].

Our results were also in contrast with Kennedy and al study [29], where significant differences in DNA yield and bacterial composition for kits used to isolate bacterial DNA from stool were observed. However, the PCR products were sequenced by the Roche 454 and not by Illumina. The lower error rates, higher throughput [30] and higher read quality [31] obtained with Illumina technology, results in higher quality data and could explain these differences.

Nucleic acid quality and quantity differed significantly not only between the DNA extraction kit method [27,32,33], but also by sample types differing in bacterial biomass. Chymes, BAL and sputum can be considered low biomass samples [34–36]. The complexities of 16S rDNA sequencing from low biomass samples are increasingly recognized and have broad applicability [34,37–40]. In our hand, none of the extraction kits tested with manufacturer conditions was efficient to extract DNA with sufficient quality and quantity for further analysis DNA extraction protocols should probably be adapted to this type of very demanding samples, by adapting the storage, the mode of lysis, the buffers used, etc. Moreover, it seems important to introduce Mock standards in these methods, to prevent possible contamination of these low biomass samples with high biomass samples (stools) that can be extracted in parallel [41].

## Conclusion

Although MPLCD was the less efficient kit in terms of DNA quantity and quality, it was as efficient as QPFD, MNS and MNT kits to capture microbial richness and diversity for stool samples. These results will prove useful for researchers or practitioners investigating the microbiota in selecting an alternative kit to an other kit or a discontinued kit. These highlight the potential comparable results when cross-comparing studies that use these different kits.

**Summary points**

- When studying the microbiota using next-generation sequencing, comparison of various study results is often difficult due to the use of different extraction methods that may have different extraction efficiency for microbes and may change the outcomes.
- In this study, we compared four commercial DNA isolation kits. QIAamp PowerFecal Pro DNA kit (QPFPD, QIAGEN), Macherey Nucleospin Soil (MNS, MACHEREY-NAGEL) Macherey Nucleospin Tissue (MNT, MACHEREY-NAGEL) and MagnaPure LC DNA isolation kit III (MPLCD, ROCHE) were used to extract DNA from various high- (feces) and low- (chyme, bronchoalveolar lavage fluid and sputum) biomass samples. We analyzed the DNA quality and quantity by Nanodrop, gel electrophoresis and Qubit and by bacterial microbiota profiling.
- All four-extraction methods showed various DNA quantity and quality performance for all samples.
- The sequencing procedure resulted in too few reads for the low-biomass samples (chyme, bronchoalveolar lavage fluid and sputum) highlighting the importance of sufficiently sensitive methods for low-biomass samples.
- Despite the differences in quality and quantity of DNAs, the four DNA extraction kits perform equally well for fecal samples in terms of diversity and taxonomy composition.
- The results from this study can help researchers or practitioners to select an appropriate DNA extraction method to obtain reliable data and final biological interpretation.

## References

[1] van de Guchte M, Blottière HM, Doré J. Humans as holobionts: implications for prevention and therapy. Microbiome. 6(1), 81 (2018). 29716650 10.1186/s40168-018-0466-8 PMC5928587

[2] Sinha R, Abu-Ali G, Vogtmann E Assessment of variation in microbial community amplicon sequencing by the Microbiome Quality Control (MBQC) project consortium. Nat Biotechnol. 35(11), 1077–1086 (2017). 28967885 10.1038/nbt.3981 PMC5839636

[3] Ezzy AC, Hagstrom AD, George C Storage and handling of human faecal samples affect the gut microbiome composition: a feasibility study. J. Microbiol. Meth. 164, 105668 (2019). 10.1016/j.mimet.2019.105668 31302202

[4] Yu Z, Morrison M. Improved extraction of PCR-quality community DNA from digesta and fecal samples. BioTechniques 36(5), 808–812 (2004). 15152600 10.2144/04365ST04

[5] Sohrabi M, Nair RG, Samaranayake LP The yield and quality of cellular and bacterial DNA extracts from human oral rinse samples are variably affected by the cell lysis methodology. J. Microbiol. Methods. 122, 64–72 (2016). 26812577 10.1016/j.mimet.2016.01.013

[6] Costea PI, Zeller G, Sunagawa S Towards standards for human fecal sample processing in metagenomic studies. Nat. Biotechnol. 35(11), 1069–1076 (2017). 28967887 10.1038/nbt.3960

[7] Santiago A, Panda S, Mengels G Processing faecal samples: a step forward for standards in microbial community analysis. BMC Microbiol. 14, 112 (2014). 24884524 10.1186/1471-2180-14-112 PMC4021188

[8] Lee J-H, Park Y, Choi JR, Lee EK, Kim H-S. Comparisons of three automated systems for genomic DNA extraction in a clinical diagnostic laboratory. Yonsei Med J. 51(1), 104–110 (2010). 20046522 10.3349/ymj.2010.51.1.104 PMC2799962

[9] Wilfinger WW, Mackey K, Chomczynski P. Effect of pH and ionic strength on the spectrophotometric assessment of nucleic acid purity. BioTechniques 22(3), 474–476 478–481 (1997). 9067025 10.2144/97223st01

[10] Verschuren LMG, Calus MPL, Jansman AJM Fecal microbial composition associated with variation in feed efficiency in pigs depends on diet and sex. J. Anim. Sci. 96(4), 1405–1418 (2018). 29669075 10.1093/jas/sky060 PMC6095354

[11] Magoč T, Salzberg SL. FLASH: fast length adjustment of short reads to improve genome assemblies. Bioinformatics. 27(21), 2957–2963 (2011). 21903629 10.1093/bioinformatics/btr507 PMC3198573

[12] Zemb O, Achard CS, Hamelin J Absolute quantitation of microbes using 16S rRNA gene metabarcoding: a rapid normalization of relative abundances by quantitative PCR targeting a 16S rRNA gene spike-in standard. Microbiologyopen. 9(3), e977 (2020). 31927795 10.1002/mbo3.977 PMC7066463

[13] Quast C, Pruesse E, Yilmaz P The SILVA ribosomal RNA gene database project: improved data processing and web-based tools. Nucleic Acids Res. 41(Database issue), D590–596 (2013). 23193283 10.1093/nar/gks1219 PMC3531112

[14] Rognes T, Flouri T, Nichols B, Quince C, Mahé F. VSEARCH: a versatile open source tool for metagenomics. PeerJ. 4, e2584 (2016). 27781170 10.7717/peerj.2584 PMC5075697

[15] Carrigg C, Rice O, Kavanagh S, Collins G, O'Flaherty V. DNA extraction method affects microbial community profiles from soils and sediment. Appl. Microbiol. Biotechnol. 77(4), 955–964 (2007). 17960375 10.1007/s00253-007-1219-y

[16] Feinstein LM, Sul WJ, Blackwood CB. Assessment of Bias Associated with Incomplete Extraction of Microbial DNA from Soil. Appl. Environ. Microbiol. 75(16), 5428–5433 (2009). 19561189 10.1128/AEM.00120-09 PMC2725469

[17] Yuan S, Cohen DB, Ravel J, Abdo Z, Forney LJ. Evaluation of Methods for the Extraction and Purification of DNA from the Human Microbiome. PLOS ONE. 7(3), e33865 (2012). 22457796 10.1371/journal.pone.0033865 PMC3311548

[18] Claassen S, du Toit E, Kaba M, Moodley C, Zar HJ, Nicol MP. A comparison of the efficiency of five different commercial DNA extraction kits for extraction of DNA from faecal samples. J. Microbiol. Meth. 94(2), 103–110 (2013). 10.1016/j.mimet.2013.05.008 PMC5809576 23684993

[19] Wu GD, Lewis JD, Hoffmann C Sampling and pyrosequencing methods for characterizing bacterial communities in the human gut using 16S sequence tags. BMC Microbiol. 10(1), 206 (2010). 20673359 10.1186/1471-2180-10-206 PMC2921404

[20] de Boer R, Peters R, Gierveld S, Schuurman T, Kooistra-Smid M, Savelkoul P. Improved detection of microbial DNA after bead-beating before DNA isolation. J. Microbiol. Meth. 80(2), 209–211 (2010). 10.1016/j.mimet.2009.11.009 19995580

[21] Gryp T, Glorieux G, Joossens M, Vaneechoutte M. Comparison of five assays for DNA extraction from bacterial cells in human faecal samples. J. Appl. Microbiol. 129(2), 378–388 (2020). 32034968 10.1111/jam.14608 PMC7384110

[22] Lim MY, Park Y-S, Kim J-H, Nam Y-D. Evaluation of fecal DNA extraction protocols for human gut microbiome studies. BMC Microbiol. 20(1), 212 (2020). 32680572 10.1186/s12866-020-01894-5 PMC7367376

[23] Griffiths L, Chacon-Cortes D. Methods for extracting genomic DNA from whole blood samples: current perspectives. BSAM. 2, 1–9 (2014).

[24] Berensmeier S. Magnetic particles for the separation and purification of nucleic acids. Appl. Microbiol. Biotechnol. 73(3), 495–504 (2006). 17063328 10.1007/s00253-006-0675-0 PMC7080036

[25] Franzreb M, Siemann-Herzberg M, Hobley TJ, Thomas ORT. Protein purification using magnetic adsorbent particles. Appl. Microbiol. Biotechnol. 70(5), 505–516 (2006). 16496138 10.1007/s00253-006-0344-3

[26] Archer MJ, Lin B, Wang Z, Stenger DA. Magnetic bead-based solid phase for selective extraction of genomic DNA. Anal. Biochem. 355(2), 285–297 (2006). 16764814 10.1016/j.ab.2006.05.005

[27] Knudsen BE, Bergmark L, Munk P Impact of Sample Type and DNA Isolation Procedure on Genomic Inference of Microbiome Composition. mSystems. 1(5), e00095–16 (2016). 27822556 10.1128/mSystems.00095-16 PMC5080404

[28] Ducarmon QR, Hornung BVH, Geelen AR, Kuijper EJ, Zwittink RD. Toward Standards in Clinical Microbiota Studies: Comparison of Three DNA Extraction Methods and Two Bioinformatic Pipelines. mSystems. 5(1), e00547–19 (2020). 32047058 10.1128/mSystems.00547-19 PMC7018525

[29] Kennedy NA, Walker AW, Berry SH The impact of different DNA extraction kits and laboratories upon the assessment of human gut microbiota composition by 16S rRNA gene sequencing. PLOS ONE. 9(2), e88982 (2014). 24586470 10.1371/journal.pone.0088982 PMC3933346

[30] Loman NJ, Misra RV, Dallman TJ Performance comparison of benchtop high-throughput sequencing platforms. Nat. Biotechnol. 30(5), 434–439 (2012). 22522955 10.1038/nbt.2198

[31] Salipante SJ, Kawashima T, Rosenthal C Performance comparison of Illumina and ion torrent next-generation sequencing platforms for 16S rRNA-based bacterial community profiling. Appl. Environ. Microbiol. 80(24), 7583–7591 (2014). 25261520 10.1128/AEM.02206-14 PMC4249215

[32] Lim MY, Song E-J, Kim SH, Lee J, Nam Y-D. Comparison of DNA extraction methods for human gut microbial community profiling. Systematic and Applied Microbiology. 41(2), 151–157 (2018). 29305057 10.1016/j.syapm.2017.11.008

[33] Klindworth A, Pruesse E, Schweer T Evaluation of general 16S ribosomal RNA gene PCR primers for classical and next-generation sequencing-based diversity studies. Nucleic Acids Res. 41(1), e1 (2013). 22933715 10.1093/nar/gks808 PMC3592464

[34] Karstens L, Siddiqui NY, Zaza T, Barstad A, Amundsen CL, Sysoeva TA. Benchmarking DNA isolation kits used in analyses of the urinary microbiome. Sci Rep. 11(1), 6186 (2021). 33731788 10.1038/s41598-021-85482-1 PMC7969918

[35] Saladié M, Caparrós-Martín JA, Agudelo-Romero P, Wark PAB, Stick SM, O'Gara F. Microbiomic Analysis on Low Abundant Respiratory Biomass Samples; Improved Recovery of Microbial DNA From Bronchoalveolar Lavage Fluid. Front Microbiol. 11, 572504 (2020). 33123104 10.3389/fmicb.2020.572504 PMC7573210

[36] Claassen-Weitz S, Gardner-Lubbe S, Mwaikono KS, du Toit E, Zar HJ, Nicol MP. Optimizing 16S rRNA gene profile analysis from low biomass nasopharyngeal and induced sputum specimens. BMC Microbiol. 20(1), 113 (2020). 32397992 10.1186/s12866-020-01795-7 PMC7218582

[37] Drengenes C, Wiker HG, Kalananthan T, Nordeide E, Eagan TML, Nielsen R. Laboratory contamination in airway microbiome studies. BMC Microbiol. 19(1), 187 (2019). 31412780 10.1186/s12866-019-1560-1 PMC6694601

[38] Dahlberg J, Sun L, Persson Waller K Microbiota data from low biomass milk samples is markedly affected by laboratory and reagent contamination. PLOS ONE. 14(6), e0218257 (2019). 31194836 10.1371/journal.pone.0218257 PMC6564671

[39] Eisenhofer R, Minich JJ, Marotz C, Cooper A, Knight R, Weyrich LS. Contamination in Low Microbial Biomass Microbiome Studies: Issues and Recommendations. Trends Microbiol. 27(2), 105–117 (2019). 30497919 10.1016/j.tim.2018.11.003

[40] Theis KR, Romero R, Winters AD Does the human placenta delivered at term have a microbiota? Results of cultivation, quantitative real-time PCR, 16S rRNA gene sequencing, and metagenomics. Am. J. Obstet. Gynecol. 220(3), 267.e1–267.e39 (2019). 10.1016/j.ajog.2018.10.018 PMC6733039 30832984

[41] Videnska P, Smerkova K, Zwinsova B Stool sampling and DNA isolation kits affect DNA quality and bacterial composition following 16S rRNA gene sequencing using MiSeq Illumina platform. Sci Rep. 9(1), 13837 (2019). 31554833 10.1038/s41598-019-49520-3 PMC6761292

